# The Sensitivity of a Specific Denitrification Rate under the Dissolved Oxygen Pressure

**DOI:** 10.3390/ijerph17249366

**Published:** 2020-12-14

**Authors:** Massimo Raboni, Paolo Viotti, Elena Cristina Rada, Fabio Conti, Maria Rosaria Boni

**Affiliations:** 1 Civil Engineering, University of Pavia, 5, 27100 Pavia, Italy; massimo.raboni@gmail.com; 2 Department of Civil and Environmental Engineering, University of Rome “LA Sapienza”, 18, 00184 Roma, Italy; paolo.viotti@uniroma1.it (P.V.); mariarosaria.boni@uniroma1.it (M.R.B.); 3 Department of Theoretical and Applied Sciences, Insubria University, 46, 21100 Varese, Italy; fabio.conti@uninsubria.it

**Keywords:** activated sludge, biological model, biological process, denitrification, dissolved oxygen, nitrogen removal, specific denitrification rate

## Abstract

The biological denitrification process is extensively discussed in scientific literature. The process requires anoxic conditions, but the influence of residual dissolved oxygen (*DO*) on the efficiency is not yet adequately documented. The present research aims to fill this gap by highlighting the effects of *DO* on the specific denitrification rate (*SDNR*) and consequently on the efficiency of the process. *SDNR* at a temperature of 20 °C (*SDNR*_20°C_) is the parameter normally used for the sizing of the denitrification reactor in biological-activated sludge processes. A sensitivity analysis of *SNDR*_20°C_ to *DO* variations is developed. For this purpose, two of the main empirical models illustrated in the scientific literature are taken into consideration, with the addition of a deterministic third model proposed by the authors and validated by recent experimentations on several full-scale plants. In the first two models, *SDNR*_20°C_ is expressed as a function of the only variable food:microrganism ratio in denitrification (*F:M_DEN_*), while in the third one, the dependence on *DO* is made explicit. The sensitivity analysis highlights all the significant dependence of *SDNR*_20°C_ on *DO* characterized by a logarithmic decrease with a very pronounced gradient in correspondence with low *DO* concentrations. Moreover, the analysis demonstrates the relatively small influence of *F:M_DEN_* on the *SDNR*_20°C_ and on the correlation between *SDNR*_20°C_ and *DO*. The results confirm the great importance of minimizing *DO* and limiting, as much as possible, the transport of oxygen in the denitrification reactor through the incoming flows and mainly the mixed liquor recycle. Solutions to achieve this result in full-scale plants are reported.

## 1. Introduction

As is known, the nitrogen contained in wastewater can be removed with physicochemical or biological processes. The most known usage of physicochemical processes consists of the removal of ammonia from industrial wastewaters by stripping [1,2,3,4,5,6,7,8,9]. Most of the other physicochemical processes are essentially based on ammonia oxidation. Currently, they are mainly confined to the research area [5,10,11,12]. By contrast, biological processes (both suspended and attached biomasses) are largely used for nitrogen removal from sewage and several industrial wastewaters due to their greater cost-effectiveness.

**Figure 1:**
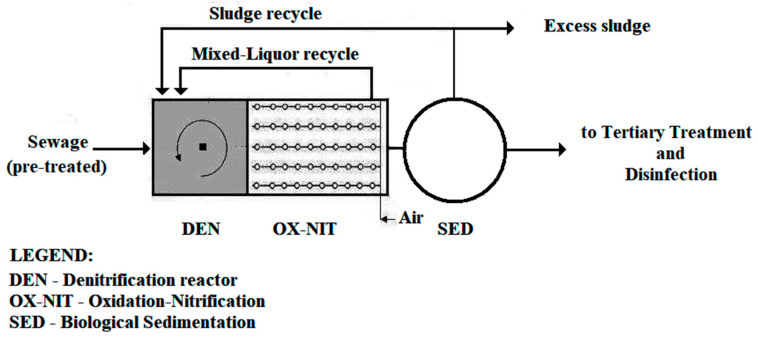
Simplified layout of a biological treatment plant with pre-denitrification [18].

Currently, biological pre-denitrification, in activated sludge processes, is the most widely used technology. As shown in Figure 1, it consists of an anoxic reactor (DEN) located upstream of the aerobic oxidizing–nitrifying reactor (OX-NIT) which is responsible for the removal of biochemical oxygen demand (BOD_5_) and for the nitrification of total Kjeldhal nitrogen (TKN) [13,14,15,16,17,18,19,20,21,22,23,24,25,26].

The pre-denitrification process is based on the activity of heterotrophic bacteria capable of reducing nitrates to nitrogen through the biochemical use of the biodegradable organic substrate present in the wastewater [18,19]. The process is very well known and is very reliable from the operational point of view. Research in this field is mainly aimed at the study of models for the optimal sizing of the denitrification reactor. Other important lines of research concern the study of the influence of various operational parameters on the process efficiency. Specifically, the most important parameters are temperature, sludge loading, residual dissolved oxygen (*DO*), hydrodynamic behavior of the reactor, the fluctuations of raw wastewater loads, and also the alternative organic matrices that can be used for the reduction of nitrates in case of particular industrial wastewaters.

In the past, the design of the biological pre-denitrification reactor was based on the knowledge of the denitrification rate at 20 °C, which was supposed to be characterized by a zero-order kinetics (for both nitrate and organic substrates). The denitrification rate at the real temperature *T* of the mixed liquor, (*r_DEN_*)*_T_* (gNO_3_-N h^−1^ kgMLVSS^−1^), was calculated by the Arrhenius equation [27]:

(1) $${\left(r_{DEN}\right)}_{T}={\left(r_{DEN}\right)}_{20{}^{\circ}C}\cdot \theta^{T-20}$$

where:

- *(r_DEN_)*_20°C_ is the denitrification rate at 20 °C, equal to 2.9 ÷ 3.0 gNO_3_-N h^−1^ kgMLVSS^−1^;
- *θ* is the temperature coefficient: *θ* = 1.026 (US-EPA, 2009); *θ* = 1.07 [28].

This criterion is still used nowadays, even if a more extended equation which also keeps in consideration the influence of both the substrates (NO_3_-N and BOD_5_) and the *DO* is preferred.

A more recent approach to the sizing of the denitrification reactor is based on the calculation of the specific denitrification rate (*SDNR*) defined as follows:

(2) $$SDNR_{T}=\;\frac{Q\cdot \Delta N}{V\cdot MLVSS}$$

(3) $$SDNR_{T}=\;SDNR_{20{}^{\circ}C}\cdot \theta^{\left(T\;-\;20\right)}$$

where:

- *SDNR_T_* is the specific denitrification rate at the temperature *T* (kgNO_3_-N·kgMLVSS^−1^·d^−1^);
- *SDNR*_20°C_ is the specific denitrification rate at the temperature of 20 °C (kgNO_3_-N·kgMLVSS^−1^·d^−1^);
- *Q·*Δ*N* is the load of nitrogen removed in denitrification (kg·d^−1^);
- MLVSS is the mixed liquor volatile suspended solids in denitrification (kgVSS·m^−3^);
- *T* is the temperature of mixed liquor (°C).

Indeed, the *SDNR_T_* is the sum of two contributions: the dissimilative denitrification (biochemical reduction of nitrate to nitrogen) and assimilative denitrification (cell synthesis). Once the value of *SDNR*_20°C_ is known, it is possible to calculate the volume of the denitrification reactor through the Equations (2) and (3).

Several models are proposed in the literature for the calculation of *SDNR*_20°C_, and most of them are empirical. These models express *SDNR*_20°C_ as a function of variables and constants which theoretically should consider all the factors influencing the denitrification kinetics [29,30,31,32,33].

As is known, the biological denitrification process requires anoxic conditions. The influence of dissolved oxygen on the efficiency of the denitrification process has been reported by several studies. Already in 1972, Dawson and Murphy [34] had found a *DO* inhibition on denitrification at *DO* concentrations of 0.20 mg/L. Other studies have evidenced the effect of *DO* inhibition [35,36,37,38]. In particular, Oh and Silverstein [38] proved a significant inhibition effect at *DO* concentration as low as 0.09 mg/L with a correspondent 35% reduction of the denitrification rate. Moreover, the United States Environmental Protection Agency, EPA, has confirmed in its reports the denitrification rate with a dependency from *DO* [39,40]. Later, the strong influence of *DO* was demonstrated experimentally on pilot plants and full-scale plants [41,42]. Despite this specific scientific information, there is currently a need to have full knowledge of the influence of *DO*, at different concentrations, on the *SDNR*_20 °C_. The present research aims to fill this gap by highlighting the sensitivity of *SDNR*_20°C_ toward *DO* in denitrification. For this purpose, three of the most effective calculation models proposed by the scientific literature have been used. Two of these models are empirical and still widely used in plant design today. The third model is more recent and noteworthy as, for the first time, it makes the dependence on *DO* explicit.

## 2. Materials and Methods

The three *SDNR*_20°C_ models on which the sensitivity analysis is based are illustrated below:

- Model I: It is described in Tchobanoglous [28]. It is very simple and consists of an empirical correlation of *SDNR*_20°C_ with only the sludge loading in DEN (*F:M_DEN_*). It was largely used for the sizing of denitrification reactors. This model does not make explicit the influence of residual *DO* in denitrification; as such, it does not consider the *DO* as a limiting factor of denitrification kinetic at the relatively small *DO* concentrations (about 0.25–0.40 mg L^−1^) that occur on well-designed and well-managed full-scale plants.
  (4) $$SDNR_{20{}^{\circ}C}=0.029+0.03\cdot F:M_{DEN}$$
- Model II: The US EPA [39] proposed a very similar equation but applied a correction factor *F_b_* to the *F:M_DEN_* in order to take into account the deviation of the active fraction of biomass in the mixed liquor from the reference value of 0.3.
  (5) $$SDNR_{20{}^{\circ}C}=0.029+0.03\;\cdot (F_{b}/0.30)\cdot \left(F:M_{DEN}\right)$$
  The correction factor *F_b_* depends on the solid retention time (*SRT*).
  Fb= {[YH(1+bT SRT)][YH(1+bT SRT) +YI]}
  where:
  *F_b_* = Active fraction of MLVSS;
  *Y_H_* = Heterotrophic biomass synthesis yield, 0.47 g VSS/g BOD_5_;
  *b_T_* = Endogenous decay rate at temperature T of mixed liquor, g VSS/g VSS d;
  *b*_20_ = Endogenous decay rate at 20 °C = 0.10 g VSS/g VSS d;
  *Y_I_* = Inert VSS fraction in the influent, g VSS inert/g BOD_5_.
  The influent inert fraction *Y_I_* can greatly influence the active biomass fraction of the MLVSS. Values for *Y_I_* generally fall in the range 0.10–0.30 for plants with primary treatment and 0.30–0.50 for plants without primary treatment [19].
  In plants with high efficiency for both nitrification and denitrification, the solid retention time can be assumed to be in the range of 18–20 days [43,44]. With *SRT* = 20, the factor is *F_b_* = 0.35.
- Model III: This model was developed by the authors on a deterministic basis [41]. It is characterized by the explicit influence of *DO* as well as of the *F:M_DEN_*. The model was first validated by a pilot plant experimentation (sewage flow rate *Q* = 1.5–2.5 m^3^·h^−1^ variable along the experimentation) and subsequently by testing eight full-scale installations of different capacity (from 10,000 eq. inhab. to 320,000 eq. inhab.) [42]. Please refer to these last two citations for each research detail that led to the elaboration and validation of the model.
  (6) $$SDNR_{20{}^{\circ}C}=0.0864\;\left(\frac{K_{O}^{\prime }}{K_{O}^{\prime }+DO}\right)+0.05\;F:M_{DEN}\;\cdot \eta_{BOD}\;\cdot \left(\frac{DO}{0.2+DO}\right)$$
  where:
  - *K′_O_ DO* inhibition constant = 0.18 mgO_2_ L^−1^;
  - *η_BOD_* = 0.85–0.95 depending on the values assumed by *F:M_DEN_*.

The experimental results obtained on the full-scale plants experimentations demonstrated the ability of the model to calculate the *SDNR*_20°C_ at any *DO* and *F:M_DEN_* values. At the same time, it is correct to note that the model has shown a modest reduction in its reliability for small capacity plants (indicatively of less than 30,000 eq. inhab.). This effect is probably determined by the greater fluctuations of the organic load fed to the plants, such as to determine a significant instability of the BOD_5_/NO_3_-N ratio in denitrification, compared to the optimal value of BOD_5_/NO_3_-N = 4. *SDNR*_20°C_ proved a very strong sensitivity to *DO*, mainly in correspondence of low *DO* concentrations (e.g., less than 0.2 mg L^−1^).

The sensitivity analysis was conducted through the graphical representation of the correlation between $SDNR_{20{}^{\circ}C}$ and *DO* for the three models. The examination of Model III was then deepened by representing it for a wide range of *DO* values. Finally, the derivative *δ*$\;SDNR_{20{}^{\circ}C}$*/*δ *DO* of this model was also checked, in order to have a direct observation of the response of $SDNR_{20{}^{\circ}C}$ to the variations of the variable *DO*.

## 3. Results and Discussion

**Figure 2:**
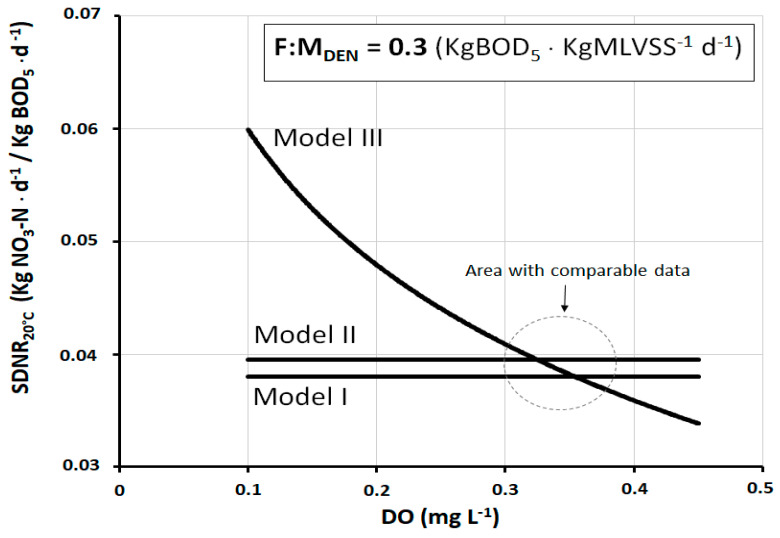
*SDNR*_20°C_ as a function of dissolved oxygen (*DO*), at *F:MDEN* = 0.3 kgBOD_5_·kgMLVSS^−1^·d^−1^, according to the three different calculation models.

Figure 2 shows the trend of *SDNR*_20°C_ as a function of *DO* in the denitrification reactor, according to the three models under study. The *F:M_DEN_* is set to 0.3 kgBOD_5_·kgMLVSS^−1^·d^−1^, a typical operating value of many full-scale plants. As Models I and II are constructed independently from *DO*, they are represented by a horizontal straight line. Instead, Model III shows a typically logarithmic trend, with a very strong gradient especially at low *DO* values (*DO* < 0.2–0.3 mg L^−1^). Then, the gradient tends progressively to reduce as *DO* increases. The inhibition effect exerted by *DO* is strongly conditioned by the phenomenon of oxygen transport from the core of the mixed liquor toward the inside of the activated sludge flocs. In the presence of small concentrations of *DO*, this phenomenon of penetration is strongly slowed down, so as to favor the creation of anoxic macrozones where the activity of denitrifying heterotrophic bacteria develops. In this sense, the great variability of *SDNR*_20°C_ at different concentrations of *DO* is partly explained.

**Figure 3:**
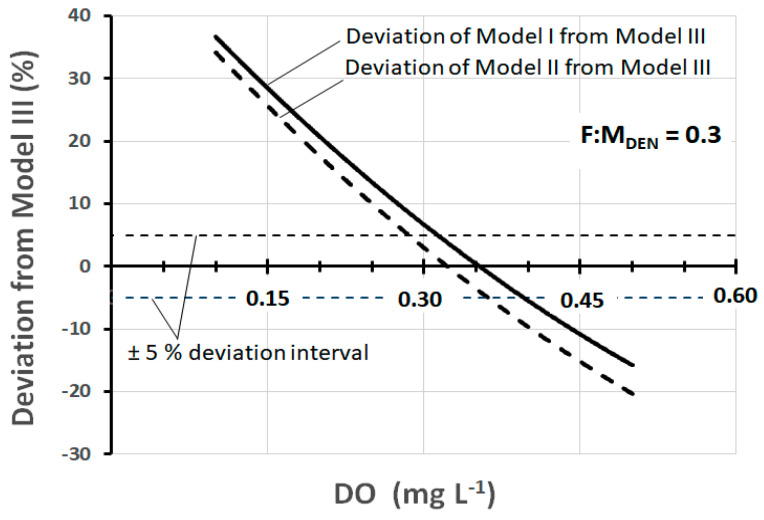
Deviation of *SDNR*_20°C_ of Models I and II from Model III, as a function of *DO*, at *F:M_DEN_* = 0.3 kgBOD_5_·kgMLVSS^−1^·d^−1.^

As can be observed, the difference between the models is considerable, but in reality, there is a range of *DO* (about 0.3–0.4 mg L^−1^) within which the values of *SDNR*_20°C_ are quite comparable. This range is better quantifiable in the graph of Figure 3 which shows the deviation of the *SDNR*_20°C_ values of Model III from those of Models I and II.

The deviation at any specific *DO* concentration is calculated as the percentage difference between *SDNR*_20°C_ of Model III and that of Models I and II. The examination of the graph allows us to observe that the deviation of ±5% corresponds to the range *DO* = 0.28–0.36 mg L^−1^ for Model II and *DO* = 0.31–0.40 mg L^−1^ for Model I.

**Figure 4:**
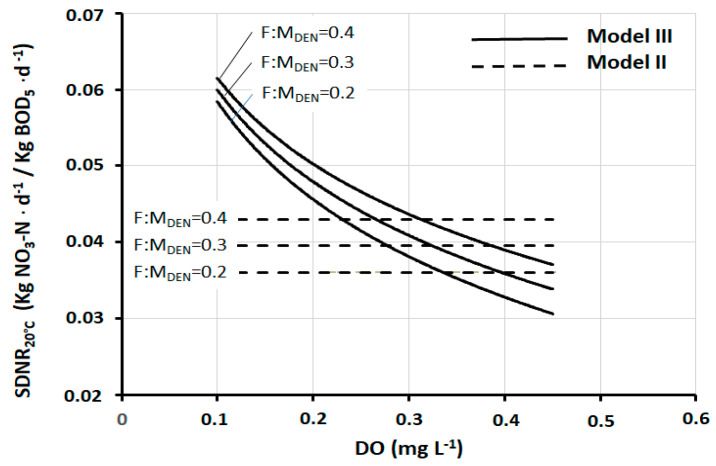
*SDNR*_20°C_ as a function of *DO*, according to Models II and III, at three different *F:M_DEN_* values.

**Figure 5:**
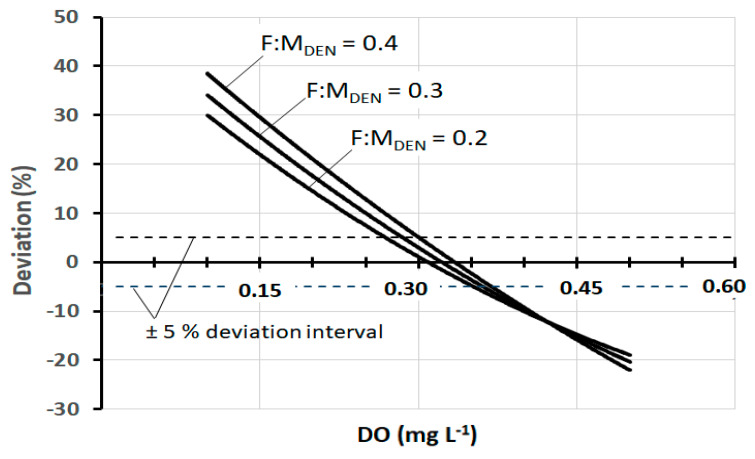
Deviation of *SDNR*_20°C_ of Model II from Model III, as a function of *DO*, at three different *F:M_DEN_* values.

In fact, in pre-denitrification reactors of full-scale plants, *DO* concentrations between 0.3 mg and 0.4 mg L^−1^ are very frequently found. Thus, within this range, the practical result produced by the three models is, in a first approximation, fairly well comparable. At the same time, it should be noted that there are well-designed and well-operated plants in which the *DO* is maintained at values slightly below 0.3 mg L^−1^, and at the same time, there are plants in which the *DO* is found at values appreciably higher than 0.4 mg L^−1^. In both cases, Models I and II lose their representativeness, while the same is preserved by Model III. Figure 4 shows the trend of *SDNR*_20°C_ as a function of *DO*, according to Models II and III (Model I is not shown for a better graphic representation) at three different values of *F:M_DEN_*. Figure 5 shows the corresponding deviation between these two models. Both graphs show that all deviation values within ±5% correspond to the range *DO* = 0.27–0.37 mg L^−1^. These data are further confirmation that the comparability of the three models exists, as a first approximation, within this specific range.

What is illustrated above highlights how relevant it is to include the *DO* in the expressions of *SDNR*_20°C_, as it is made in Model III.

**Figure 6:**
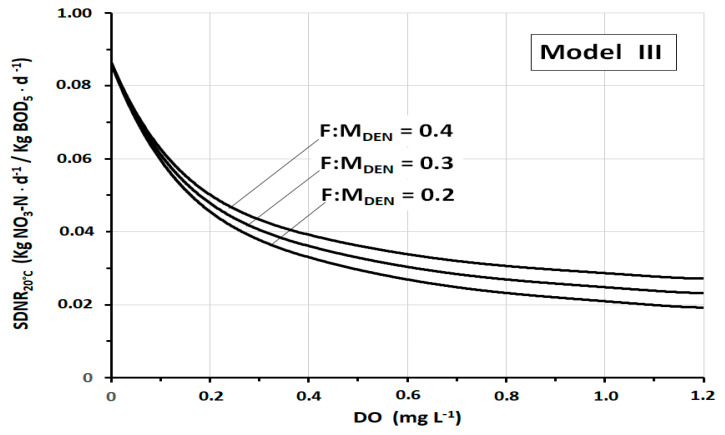
*SDNR*_20°C_ as a function of *DO* (in the wide range *DO* = 0–1.2 mg L^−1^), according to Model III, at three different *F:M_DEN_* values.

The strong influence of *DO* on *SDNR*_20°C_ is well shown in Figure 6, which illustrates the trend of *SDNR*_20°C_ of Model III as a function of the *DO* at three different values of *F:M_DEN_*. The specificity of this figure consists in the wide range of *DO* considered, from *DO* = 0 mg L^−1^ to *DO* = 1.2 mg L^−1^. At *DO* = 0 mg L^−1^, all the curves converge to the ideal value *SDNR*_20°C_ = 0.864 kgNO_3_-N·kgMLVSS^−1^·d^−1^. By contrast, at high concentrations of *DO*, the curves tend to gradually flatten, making the variations of *SDNR*_20°C_ less and less noticeable. The initial strong gradient leads to an about 45% reduction in *SDNR*_20°C_ by increasing the dissolved oxygen from *DO* = 0 mg L^−1^ to *DO* = 0.2 mg L^−1^. A further increase in *DO* to 0.4 results in a further 18% reduction of *SDNR*_20°C_. At higher *DO* values (*DO* > 1 mg L^−1^), the *SDNR*_20°C_ tends to be determined more and more by only cellular synthesis. The graph also shows how, although appreciable, the incidence of *F:M_DEN_* is relatively limited.

**Figure 7:**
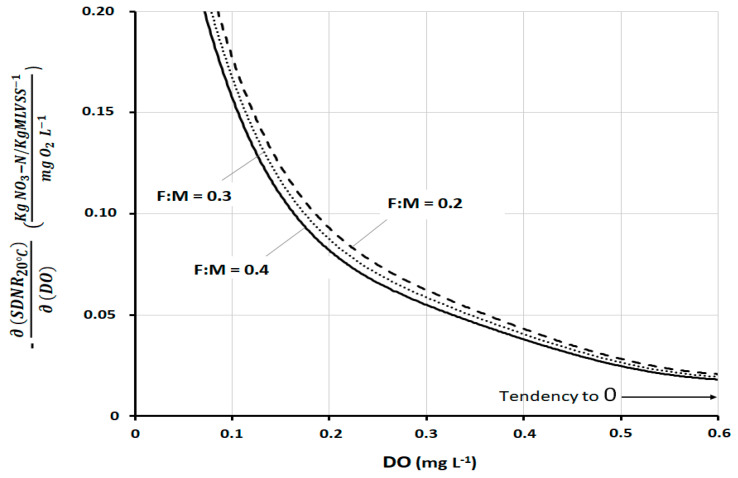
Derivative of *SDNR*_20°C_ with respect to *DO*, according to Model III at three different *F:M_DEN_*_._

A full confirmation of the above evaluations can be seen in Figure 7, which shows the mathematical derivative of Model III, always as a function of *DO*. The derivative expresses the response of *SDNR*_20°C_ to the variation of each specific *DO* value. As such, it expresses the sensitivity better than any other parameter. The curves show a very high sensitivity of *SDNR*_20°C_ to *DO*, mainly at low *DO* concentrations (*DO* approximately < 0.3 mg L^−1^). The sensitivity then tends to decrease with a progressively less pronounced gradient with increasing *DO* values (above 0.4–0.5 mg L^−1^). It is useful to consider that *DO* greater than 0.5–0.6 mg L^−1^ is rarely found on well-designed and operated full-scale plants. This figure, even more so than the previous one, shows that the variations of *F:M_DEN_* are of little relevance and do not appreciably influence this sensitivity analysis.

Overall, the results of the present sensitivity analysis highlight the need to limit, as much as possible, the concentration of *DO* in the denitrification reactor, and this should already be done at the plant design stage. Among the measures that can be adopted, the following can be mentioned:

- An appropriate hydrodynamic configuration for the denitrification reactor. In fact, the results of specific experimentations highlight the influence of the hydrodynamic model of the anoxic reactor on the residual *DO* and consequently on the denitrification efficiency. A set of four reactors demonstrates greater oxygen consumption capability (allowing residual *DO* below 0.1 mg L^−1^) than a single complete mixing reactor (allowing residual *DO* of 0.18–0.30 mg L^−1^). It is therefore necessary to provide configurations of the denitrification reactor tending as much as possible to the plug-flow [45].
- A post-anoxic reactor (after the pre-denitrification and oxidation-nitrification steps) proved to be very effective in improving the efficiency of nitrogen removal [46]. In fact, in this stage, a marked reduction of the dissolved oxygen can be obtained so as to allow the recycling of the mixed liquor with a relatively small *DO*. The reduction of the dissolved oxygen in the post-anoxic reactor is influenced by the retention time and the sludge loading of the biological process. It was observed that, with *F:M* = 0.130 kg BOD_5_∙d^−1^∙kg MLVSS^−1^ (referring to the volumes of denitrification plus oxidation-nitrification) and 1.5 h of retention time, it is possible to obtain the reduction of the dissolved oxygen in the post-anoxic reactor to the average concentration of 0.31 mg L^−1^, such as to determine an average concentration down to 0.11 mg L^−1^ in the pre-denitrification reactor. The consumption of *DO* in the post-anoxic reactor is due to the bio-assimilation of the residual BOD and the bio-oxidation of the endogenous carbon.
- The limitation of *DO* in the inlet flows to the denitrification reactor (pre-treated sewage, sludge recycle, mixed liquor recycle) using closed pipes instead of open channels [47,48].
- The dosage of a reducing agent such as Fe^2+^ in denitrification (or in the post-anoxic reactor), which easily oxidizes to Fe^3+^, by consuming *DO*. A specific experimentation proved that dosages of 6 mg L^−1^ Fe^2+^ can lower the average *DO* concentration from 0.45 mg L^−1^ to 0.28 mg L^−1^, consequently increasing the denitrification efficiency. In addition, ferrous ion has been found to act as a catalyst for the reduction of nitrates [49,50,51].

This solution can be of particular interest in cases where the sewage treatment includes the removal of phosphorus, which would be precipitated as ferric orthophosphate [52,53].

- The careful choice of the mixed liquor stirring system (and of the relative power) in the denitrification reactor to avoid excessive surface turbulence with consequent excessive dissolution of atmospheric oxygen. In general, completely submerged agitators equipped with a power regulator are preferred. The specific power input must be limited to the minimum necessary to keep activated sludge in suspension, and it generally ranges from 8 to 12 W m^−3^ [38,39].
- The coverage of the reactor surface with floating plastic elements in order to limit the exchange of oxygen with the overlying atmosphere.

## 4. Conclusions

The sensitivity analysis highlights the strong dependence of the *SDNR*_20°C_ on dissolved oxygen (*DO*) in biological denitrification. Therefore, every reliable computational model should include this dependency. According to the following model proposed by the authors and validated by numerous experimentations on pilot plants and full-scale plants, the sensitivity proves to be particularly relevant at low values of *DO*.

(7) $$SDNR_{20{}^{\circ}C}=0.0864\left(\frac{K_{O}^{\prime }}{K_{O}^{\prime }+DO}\right)+0.05\;F:M_{DEN}\cdot \eta_{BOD}\cdot \left(\frac{DO}{0.2+DO}\right)$$

(8) $$SDNR_{T}=\;SDNR_{20{}^{\circ}C}\;\;\theta^{\left(T-20\right)}$$

where:

- *SDNR*_20°C_ = specific denitrification rate at 20 °C (kg NO_3_-N·kg MLVSS^−1^·d^−1^);
- *SDNR_T_* = specific denitrification rate at the real temperature *T* (°C) of the mixed liquor (kg NO_3_-N·kg MLVSS^−1^·d^−1^);
- *F:M_DEN_* = food:microrganism ratio in denitrification (kg BOD_5_·kg MLVSS^−1^);
- *K’_O_* = 0.18 mgO_2_ L^−1^;
- *η_BOD_* = 0.85–0.95 depending on the values assumed by *F:M_DEN_*;
- *θ* = 1.026–1.07 temperature coefficient.

In particular, the strong initial gradient leads to a reduction of about 45% of the *SDNR*_20°C_ while increasing the initial concentration *DO* = 0 mg L^−1^ up to *DO* = 0.2 mg L^−1^. A further increase to 0.4 mg L^−1^ determines an additional 18% decrease in *SDNR*_20°C_. The influence of the *F:M_DEN_* on this sensitivity analysis appears very modest. In full-scale plants, the residual concentration of *DO* in denitrification is frequently found in the range of 0.3–0.4 mg L^−1^. In some cases, this range is increased to 0.5–0.6 mg L^−1^, thus often leading to a reduced efficiency of biological denitrification. In particularly favorable cases, *DO* is found in the range of 0.2–0.3 mg L^−1^, which allows a better denitrification performance.

The objectives of a good design of the denitrification reactor must include several measures capable of limiting the *DO* as much as possible. The main measures are summarized below:

- Designing the denitrification reactor with a hydrodynamic configuration closer to plug-flow than to complete mixing;
- Minimizing the transport of oxygen with the flows entering the reactor: use of pipes instead of open channels; tapered aeration in oxidation-nitrification or even the addition of a real post-anoxic reactor;
- Dosage in denitrification (or in the eventual post-anoxic reactor) of an easily oxidizable reagent by dissolved oxygen: Fe^2+^ (i.e., as ferrous sulphate) has proven to be effective;
- Minimizing the surface oxygen exchange by the careful choice of the agitation system.

Future research developments in this field are expected, in particular regarding the sensitivity of *SDNR* with respect to *F:M_DEN_* and to the temperature.
